# *Ilnacorahenryi*, a new species of plant bug from Mexico (Heteroptera, Miridae, Orthotylinae, Orthotylini)

**DOI:** 10.3897/zookeys.796.21285

**Published:** 2018-11-15

**Authors:** Michael D. Schwartz

**Affiliations:** 1 Research Associate, Division of Invertebrate Zoology, American Museum of Natural History, New York, NY 10024, USA American Museum of Natural History New York United States of America

**Keywords:** *
Ilnacora henryi
*, Mexico, taxonomy

## Abstract

A new species of the plant bug genus *Ilnacora*, tribe Orthotylini, is described from Mexico. This species, unlike any other in the genus, is characterized by a predominantly black coloration, the absence of black scale-like setae on the pronotal disk, and unique male genitalia.

## Introduction

**Figure 1.**
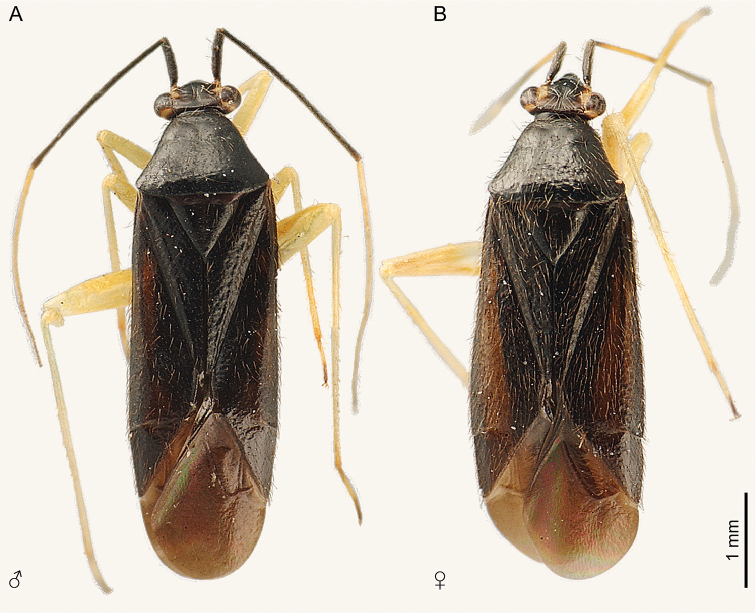
Dorsal habitus of *Ilnacorahenryi*. **A** male, AMNH_PBI 00093267 **B** female, AMNH_PBI 00093269.

**Figure 2.**
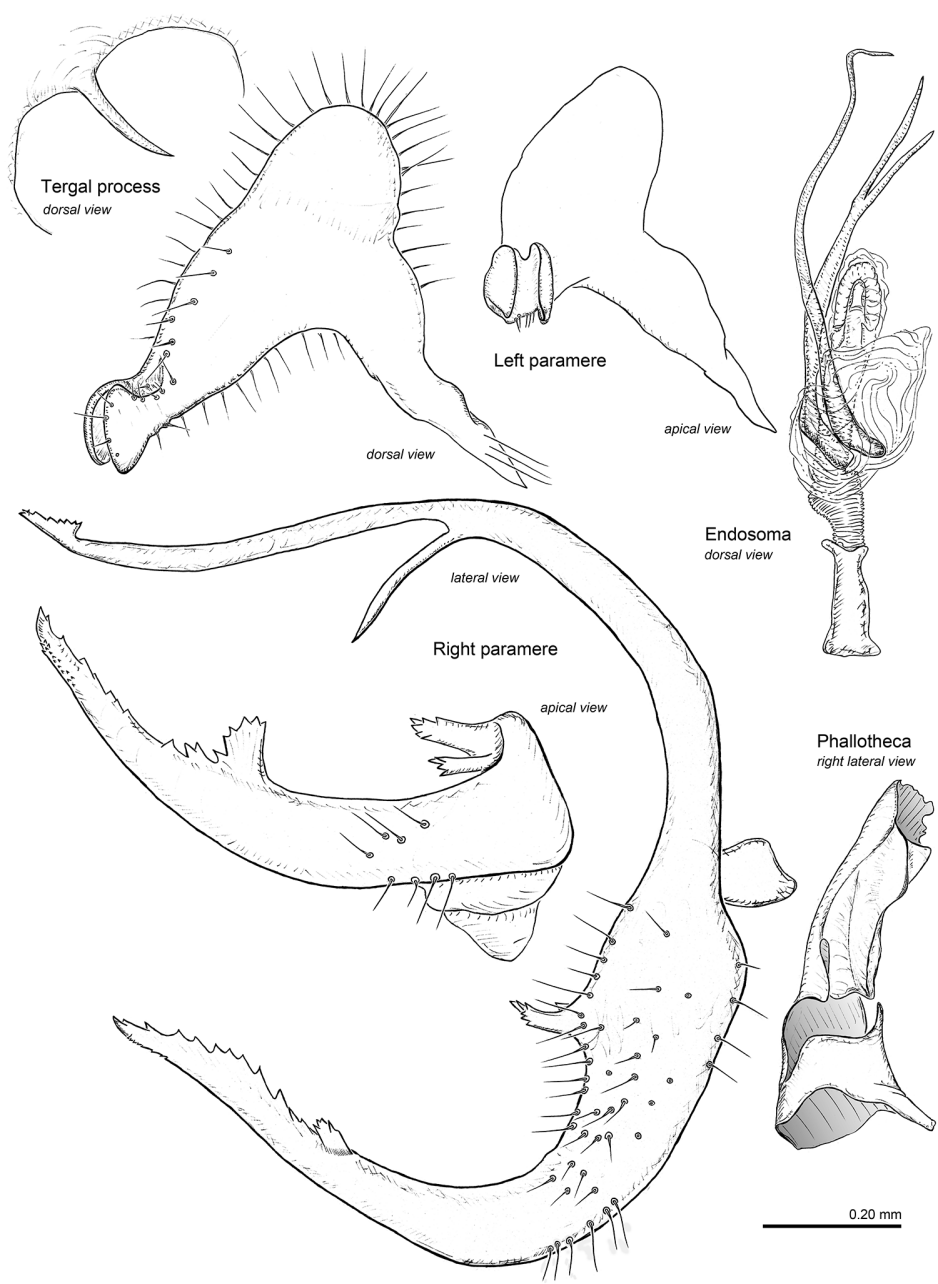
Male genitalia of *Ilnocorahenryi*.

**Figure 3.**
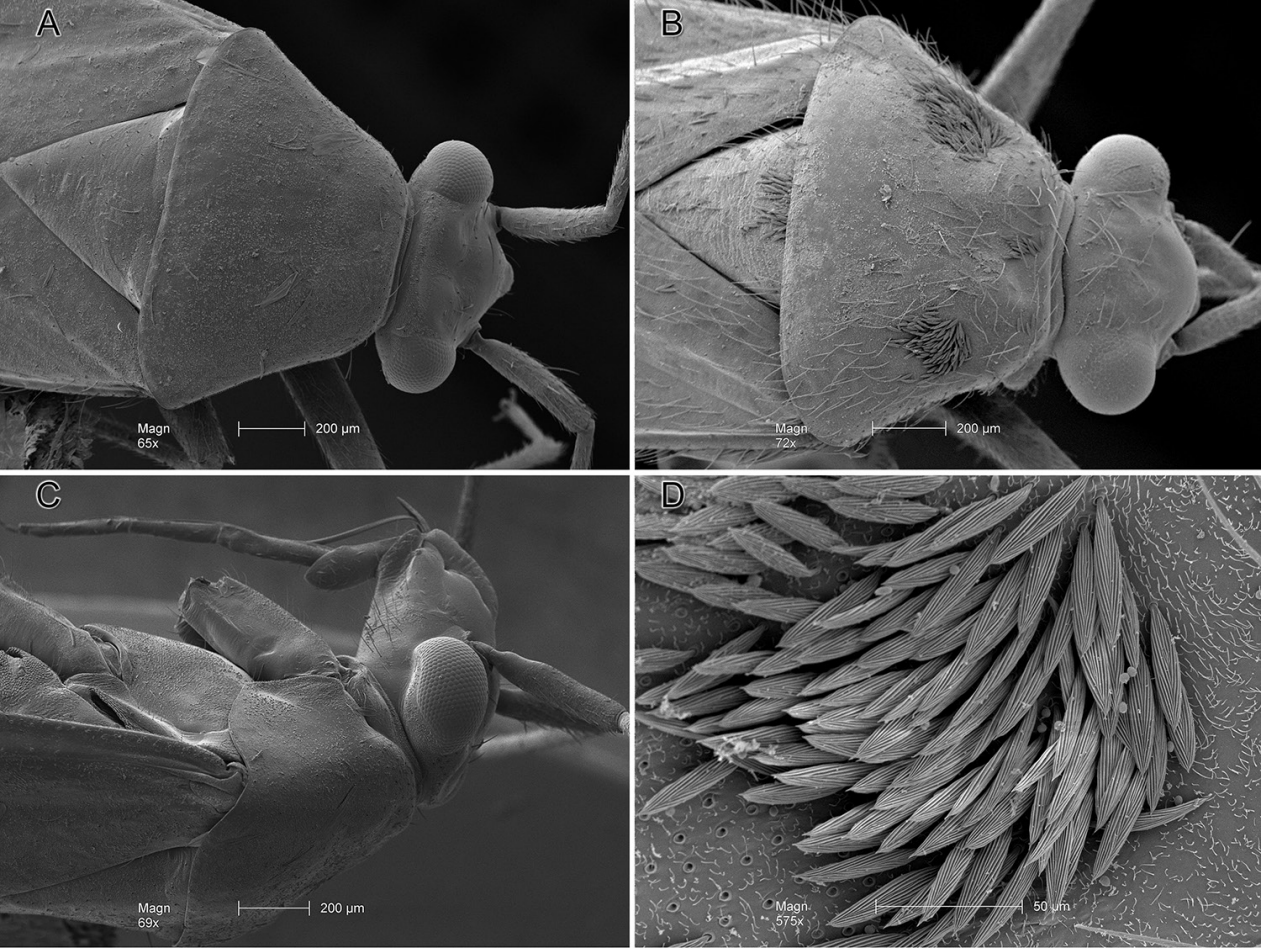
Scanning electron micrographs. **A***Ilnacorahenryi*, head and pronotum, dorsal view **B***Ilnacorainusta*, head and pronotum, dorsal view **C***Ilnacorahenryi*, head and pronotum, lateral view **D***Ilnacorainusta*, detail of pronotal scale-like setae, dorsal view.

**Figure 4.**
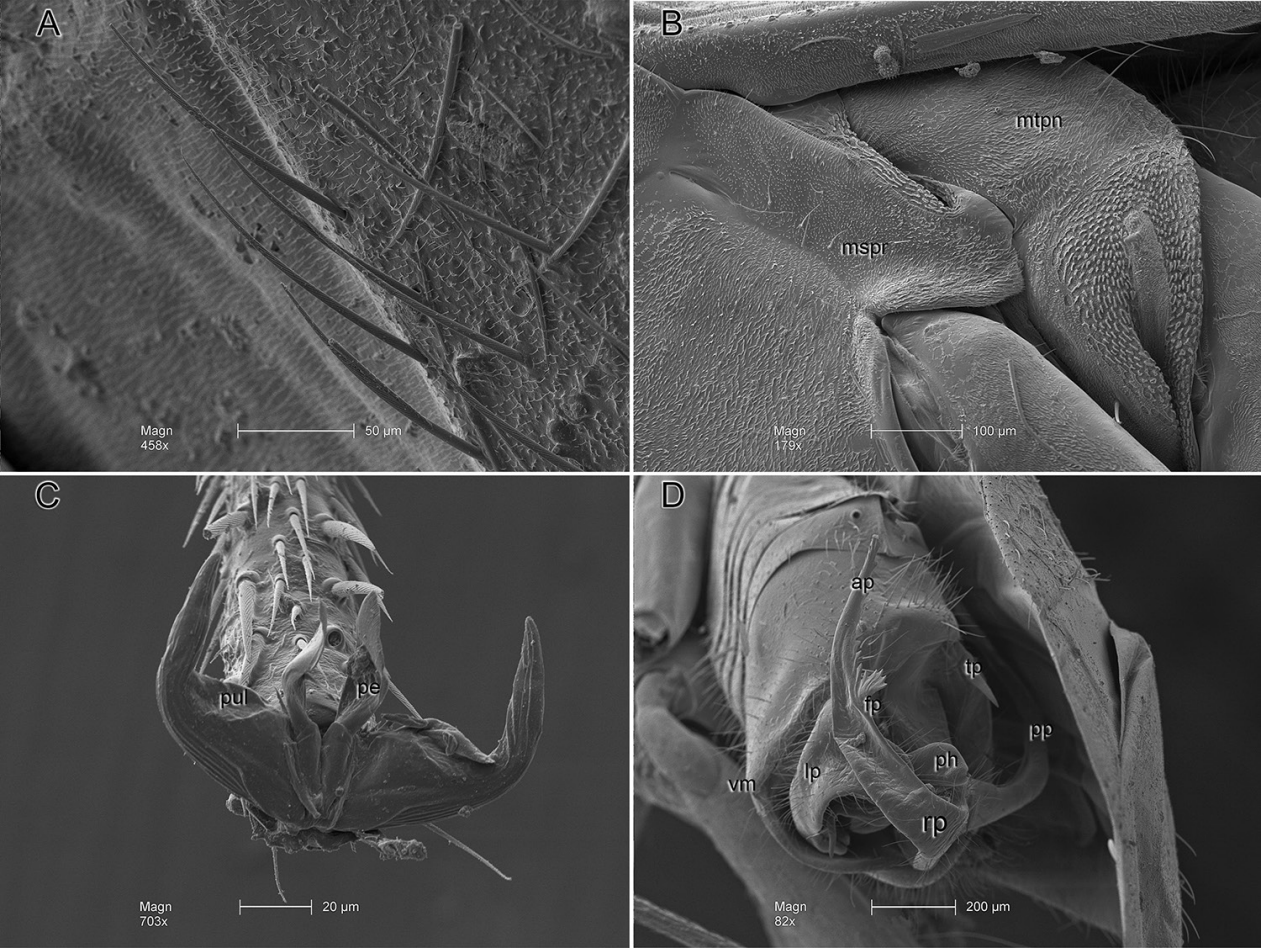
*Ilnacorahenryi*, scanning electron micrographs. **A** simple setae on edge of cuneus, lateral view **B** mesothoracic spiracle and metathoracic scent-efferent system, lateral view **C** pretarsus, frontal view **D** male genitalia, caudal view. Abbreviations: tp, tergal process; lp, left paramere; mspr, mesepimeron; mtpn, metepisternum; pe, parempodium; pul, pulvillus; rp, right paramere (ap – anterior process, fp – fan-shaped spine, pp – posterior process); ph, phallotheca; vm, ventral margin of pygophore.

A group of North American Orthotylini genera share predominately black coloration, continuous and straight posterior margin of the eyes and head, left paramere with mitten-shaped apex, and one endosomal spicule with variable arrangement and number of spines (Schaffner and Schwartz 2008; Schwartz 2011). Among the black specimens assembled for these studies were some reminiscent of *Jornandescruralis* Distant, 1893 and *J.genetivus* (Distant, 1884) but lacked the shagreen dorsal sculpturation of this genus (Fig. 1), possessed two endosomal spicules (Fig. 2), and large right paramere with long processes extending beyond the pygophore margins (Figs 2, 4D). The elaborate structure of the right paramere and presence of a tergal process is similar to that of some species of the North American genus *Ilnacora* Reuter, 1876, but the pronotum of this puzzling plant bug does not have the characteristic pair of black spots composed of black scale-like setae on the posterior pronotal disk as in *Ilnacora* (Fig. 3B, D). However, considering that the genitalia of both sexes are well within the variation encountered in the genus, I take this opportunity to describe it as a new plant bug species in this Festschrift celebrating the entomological career of Thomas J. Henry.

## Materials and methods

Data for the 50 specimens examined for this study were captured using the Arthropod Easy Capture database. All specimens bear a unique specimen identifier (USI) in the form AMNH_PBI 08011948; this alphanumeric is included on the USI label also in the form of a matrix code. For clarity the prefix is included for the holotype only. Specimen data can be viewed on-line through Discoverlife.org (http://research.amnh.org/pbi/heteropteraspeciespage) and through the iDigBio web portal.

Habitus images were prepared using a Microptics/Visionary Digital photomicrographic system as developed by Roy Larimer. Multiple layers were stacked to produce the final high-depth-of-field image using Helicon Focus software. Genitalic illustrations were initially prepared as pencil drawings using a Nikon Optiphot compound microscope and camera lucida at magnifications of 100× or 200×, then scanned and rendered as graphics using Adobe Illustrator. Photographic images of female genitalic structures temporarily placed under a coverslip in shallow well-slides containing 85% lactic acid were taken with a 10× or 20× objective lens using a Nikon E800 compound microscope, photomicrographic attachment, and software. As many as 50 layers were stacked to produce a composite high-depth-of-field image. Scanning electron micrographs of gold-coated preparations were taken with a digital Philips XL30 ESEM. The distribution map was created using SimpleMappr (Shorthouse 2010).

Measurement data presented in Table 1 include numbers of specimens measured, means, standard deviations, and ratios; all data are in millimeters. The data were captured using an ocular micrometer. Terminology of the male genitalia follows Schaffner and Schwartz (2008) and Schwartz (2011).

Specimens examined during this study came from the following collections (preceded by an institutional abbreviation) or are deposited in them followed by the names of individuals who assisted with the loan of specimens.

**AMNH**American Museum of Natural History, New York; Randall T. Schuh

**CNC**Canadian National Collection of Insects, Ottawa; Robert G. Foottit

**IBUNAM**Instituto de Biología, Universidad Nacional Autónoma de Mexico, Mexico City, D. F.; Harry Brailovsky A.

**TAMU**Department of Entomology, Texas A&M University; College Station, Texas; Joseph C. Schaffner, Edward G. Riley

**USNM**United States National Museum of Natural History, Smithsonian Institution, Washington, DC; Thomas J. Henry

## Taxonomy

### Ilnacora henryi sp. n.

Taxon classificationAnimaliaHemipteraMiridae

http://zoobank.org/6FBBD9BA-EDBB-4C7E-867C-E8968DB9A7E6

**Figure 5.**
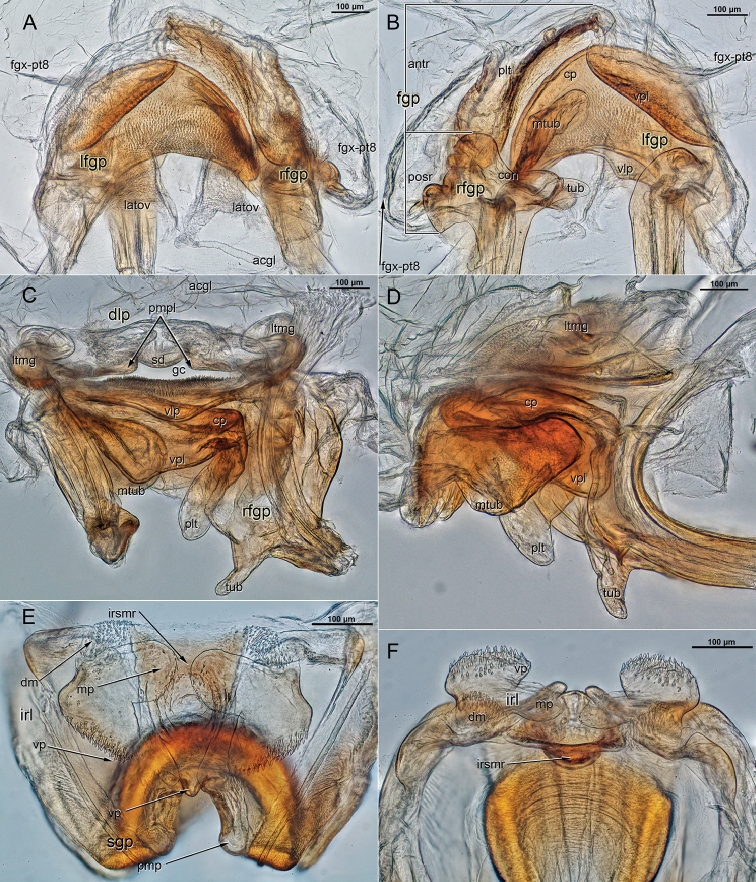
Digital female genitalic images of *Ilnacorahenryi*. **A** bursa copulatrix, dorsal view **B** bursa copulatrix, ventral view **C** bursa copulatrix, anterior view **D** bursa copulatrix, left lateral view **E** posterior wall, anterior view **F** posterior wall dorsal view. Abbreviations: acgl, accessory (vermiform) gland; dlp, dorsal labiate plate (pmpl - paramedial plate, sd – shield shaped depression; ltmg – lateral margin); fgp, first gonapophysis (antr – anterior region, posr – posterior region); fgx-pt8, membrane from first gonocoxae and fused paratergites 8; irl, interramal sclerite (dm - dorsal margin, mp - medial portion, vp - ventral portion); irsmr, interramal sclerite medial region; latov, lateral oviduct; lfgp, left first gonaphophysis (cp – crescent-shaped process, vpl – ventral plate, mtub – medial tubercle); rfgp, right first gonaphophysis (tub – tubercle, con – condyle, plt – plate-like sclerite); sgp, second gonapophysis (pmp – paramedial projection, vp – ventral projection); vlp, ventral labiate plate.

**Figure 6.**
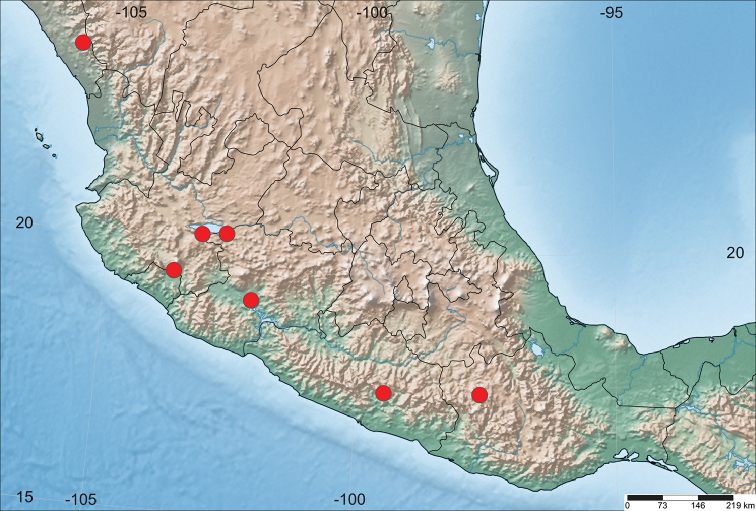
Distribution of *Ilnacorahenryi*.

Figs 1 , 2 , 3A, C , 4 , 5 , 6

#### Diagnosis.

Distinguished from congeners by practically smooth, uniformly black body with yellow legs and antennal segments 3 and 4 (Fig. 1); absence of black scale-like setae on pronotal disk posteriad of calli (Fig. 3A); elongate somewhat narrow anteocular portion of head with weakly rounded frons (Fig. 3A), transversely concave vertex and carinate posterior margin (Fig. 3A, C); mesepimeron ventrally and metepisternum with obvious microtrichia (Fig. 4B). Unequivocally recognized by unique structure of male genitalia especially narrow mostly straight tergal process, broad sensory lobe of left paramere, and three long apically serrate processes of right paramere (Figs 2, 4D).

#### Description.

*Male*: Macropterous, length 4.30–5.00 mm, width 1.40–1.65 mm (see Table 1); body moderately large, elongate.

***Coloration***: Black, except yellow on frons adjacent to medial margin of eyes, posterior margin of eyes, antennal segment 3, labial segment 2–3, and legs; variably dusky yellow to black on antennal segment 4, labial segment 4, tarsomere 3, and claw (Fig. 1).

***Vestiture and dorsal sculpture***: Sparsely distributed, long erect or reclining dark brown to black simple setae (Figs 1, 3A, 4A). Faintly rugose, without punctures (Figs 1, 3A).

***Structure***: *Head* (Figs 1, 3A, C): Strongly projecting, narrow in frontal view, gena broadly exposed, bucculae short, one-half length of labial segment 1; in lateral view (Fig. 3C); eyes small, posterior margin in dorsal view, slanted anteriad, removed from anterior margin of pronotum by diameter of antennal segment 1 (Fig. 3A), in lateral view occupying two-fifth head height of head; interocular space slightly more than twice as wide as dorsal width of eye; posterior margin of head carinate; antenna inserted just below ventral margin of eye, eyes very weakly emarginate dorsad of fossa; antennal segment 2 long (1.72 mm), 2 times width of head; labium reaching apex of mesocoxa. *Thorax*: Mesothoracic spiracle and metathoracic scent-efferent system with obvious microstructure surrounding openings; mesepimeron and metepisternum with microspicules on ventral margins (Fig. 4B). Pronotum in dorsal view subtriangular practically campanulate, lateral margin slightly concave, posterior margin gently convex; calli clearly demarcated, posterior lobe flat, rounded laterally; mesoscutum moderately exposed (Fig. 3A, C). *Pretarsus*: Claws medium sized, sharply curved, thickened proximally; parempodia wide, lamelliform, with converging apices; pulvilli fleshy, attached proximally on ventral surface of claw (Fig. 4C). *Hemelytron*: Elongate, parallel-sided, paracuneus depressed, cuneus deflected.

**Table 1.** Measurements for *Ilnacorahenryi*.

		Length				Width				Antennal length				Ratio			
		Total Body	Pronotum	Cuneus	Labium	Body	Pronotum	Head	Vertex	I	II	III	IV	HW/AII	VW/HW	HW/PW	AIII/AII
Male (N = 10)	Mean	4.65	0.84	0.70	1.32	1.52	1.28	0.84	0.44	0.46	1.72	1.39	0.89	0.49	0.53	0.65	0.81
	SD	0.21	0.02	0.02	0.03	0.07	0.03	0.01	0.01	0.02	0.09	0.13	0.07	0.02	0.01	0.01	0.08
	Range	0.70	0.09	0.05	0.10	0.25	0.13	0.04	0.04	0.06	0.31	0.50	0.19	0.07	0.03	0.05	0.26
	Min	4.30	0.81	0.68	1.28	1.40	1.20	0.82	0.43	0.44	1.54	1.13	0.81	0.47	0.51	0.64	0.62
	Max	5.00	0.90	0.73	1.38	1.65	1.33	0.86	0.46	0.50	1.85	1.63	1.00	0.53	0.54	0.68	0.88
Female (N = 10)	Mean	4.72	0.87	0.68	1.35	1.66	1.33	0.87	0.46	0.47	1.70	1.44	0.87	0.51	0.53	0.65	0.85
	SD	0.17	0.02	0.02	0.05	0.08	0.03	0.02	0.01	0.01	0.04	0.06	0.02	0.01	0.01	0.01	0.04
	Range	0.55	0.08	0.05	0.13	0.23	0.10	0.06	0.03	0.01	0.15	0.15	0.09	0.04	0.04	0.04	0.12
	Min	4.50	0.84	0.65	1.28	1.58	1.30	0.84	0.45	0.46	1.65	1.38	0.81	0.49	0.51	0.63	0.81
	Max	5.05	0.91	0.70	1.41	1.80	1.40	0.90	0.48	0.48	1.80	1.53	0.91	0.53	0.56	0.67	0.92

***Genitalia***: *Pygophore*: Dorsal margin of aperture with single, long, thin, marginally smooth, slightly curved, pointed tergal process, located just left of midline; ventroposterior margin of pygophore subquadrate, entire (without cleft) (Fig. 4D); subgenital plate raised dorsal to ventroposterior margin of aperture, forming deep cavity, ventral surface deeply notched with prominent posterior lobes—right side twice as large as left—projecting beyond aperture of pygophore posteriorly. *Left paramere*: Approximately L-shaped in ventral view; sensory lobe large, gently rounded; paramere gradually narrowed to subapical constriction, expanded to mitten-like apex formed by lateral and medial lobes of approximately equal size (Figs 2, 4D). *Right paramere*: Large, U-shaped, greatly extending beyond aperture of pygophore; posterior process (sensory lobe) as long as remainder of paramere, with fine needle-like subapical spine and slightly expanded serrate apex; middle of paramere with pair of relatively short, apically serrate lobes; anterior process (apical portion) of paramere long, distal one-half of dorsal surface serrate, subtended by fan-shaped spine (Figs 2, 4D). *Phallotheca*: Small, tubular, dorsal surface gently convoluted; aperture open distally (Figs 2, 4D). *Endosoma*: Small; formed by two needle-like spicules attached to membrane dorsal to base of ductus seminis; dorsal spicule gently curved, ventral spicule bifurcate with long, narrow spines (Fig. 2).

***Female***: Coloration, vestiture, and structure as in male, except body moderately larger, widest across cuneal fracture, costal margin slightly more convex, vertex wider, and antennal segment 2 pale yellow medially, narrower proximally; length 4.50–5.05 mm, width 1.58–1.80 mm (Fig. 1).

***Genitalia***: *Posterior margin of sternite 7*: Broadly triangular posteriorly directed flap-like projection, either side of projection incised anteriad. *Vestibulum*: First gonocoxae and fused paratergites 8 adhered to anterior surface of first gonapophyses (Fig. 5B); first gonapophyses with obvious posterior (attached to base of rami) and anterior (forming interior of vestibulum) regions (Fig. 5B, D). *First gonapophyses: Right*: Posterior region with narrow tubercle projecting across aperture of vulva (Fig. 5B–D) and with condyle-like anterior surface adjoining anterior region of right gonapophysis and apex of left first gonapophyses (Fig. 5B); anterior region narrow, plate-like (Fig. 5A–B). *Left*: Posterior region simple, smaller than right (Fig. 5B); anterior region with large crescent-shaped process in horizontal plane (Fig. 5A–B) and ventrally projecting plate laterad (Fig. 5B–C) and flat tubercle mediad (Fig. 5B). *Ventral labiate plate*: Strongly sclerotized with microspiculate dorsal surface (Fig. 5A), ventral surface broadly projecting into vulva (Fig. 5C). *Dorsal labiate plate*: Subrectangular, twice as wide and long, weakly sclerotized; paramedial sclerites microspiculate, separated at midline by membranous shield-shaped depression; lateral margins strongly infolded; sclerotized rings placed within lateral fold of dorsal labiate plate, obscure (Fig. 5A–C). *Second gonapophyses*: Anteroproximal surface projecting ventrad at midline, flanked by small round paramedial projections (Fig. 5E–F). *Posterior Wall: Interramal sclerite*: Thinly membranous, dorsomedial margin flat. *Medial region*: Strongly sclerotized, plate-like, broadly projecting posteriad abutting ovipositor bulb (Fig. 5F). *Interramal lobes*: Weakly sclerotized, broadly V-shaped, lateral surface of ventral projections and dorsal margins strongly microspiculate, middle projections sparsely microspiculate, practically meeting on midline (Fig. 5E–F).

#### Etymology.

Named to honor Dr. Thomas J. Henry for his considerable contributions to hemipteran systematics over a long, active career.

#### Hosts.

Unknown.

#### Distribution.

Known from seven widely scattered localities spanning the southern foothills of the Sierra Madre Occidental in southern Sinaloa to the western Sierra Nevada in Michoacan and east across the Sierra Madre del Sur from Colima to Oaxaca (Fig. 6).

#### Discussion.

Several congeners of *I.henryi* in the U.S. and Mexico have male genitalia of similar form. All are easily denoted by the very elongate sensory lobe of the right paramere (Knight 1963, figs 1–4, 11, 13; Knight and Schaffner 1976, figs 1, 3; Carvalho and Costa 1992, figs 4, 8). All these species also share nongenitalic characters not found in *I.henryi*: generally yellowish to green coloration with major portions of the head, pronotum and hemelytron black; pronotal disk, and sometimes scutellum and hemelytron with tufts of black scale-like setae; and head with strongly convex or tumid frons. As presented in the diagnosis and description above, the overall black body with legs yellow, absence of setal patches on the pronotal disk, and only moderate curvature of the frons make *I.henryi* unique among the species of *Ilnacora*. The new species brings to 25 the number of species composing *Ilnacora*.

Only four species, *I.inusta* (Distant, 1884), *I.mexicana* Knight & Schaffner, 1976, *I.schaffneri* Knight, 1963, and *I.tepicensis* Carvalho & Costa, 1992, are distributed within the range of *I.henryi*. The coloration of all these sympatric species is generally greenish with various small or large areas of diffuse dark color and discrete patches of black scale-like setae on scattered regions of the dorsum; the almost entirely black *I.henryi* would not be mistaken for any of these other taxa.

The majority of host associations for other species of *Ilnacora* are in Asteraceae. The following probable asteraceous hosts are recorded in the Arthropod Easy Capture database: *Ambrosia* sp., *A.trifida* L., *Artemisia* sp., Chrysopsisvillosavar.hispida (Hook.) A. Gray ex D.C. Eaton, *Coreocarpus* sp., *Dyssodiapapposa* (Vent.) Hitchc., *Ericamerianauseosa* (Pall. ex Pursh) G.L. Nesom & G.I. Baird, *Grindelia* sp., *G.hirsutula* Hook. & Arn., *G.perennis* A. Nelson, *Helianthus* sp., *Helianthussalicifolius* A. Dietr., *H.tuberosus* L., *Heterothecacanescens* (DC.) Shinners, *H.villosa* (Pursh) Shinners, *Ivaaxillaris* Pursh, *Parthenium* sp., *Solidago* sp., *S.rugosa* Mill.

**Type material.** Holotype ♂: **MEXICO: Sinaloa**: “Santa Lucia [23.49755°N, 105.92295°W], Sin. MEX. 4000' [1219 m] 4 Aug. 1964 L.A. Kelton”, (AMNH_PBI 00112931). Holotype *Ilnacorahenryi* n. sp. det. M. D. Schwartz, 2010 [red label]. Deposited in the collection of the Instituto de Biologia, Universidad Nacional Autonoma de Mexico, Mexico City, D.F. Paratypes: **MEXICO: Colima**: 9 mi NE of Comala, 19.40916°N, 103.65196°W, 18 Jul 1983, Kovarik, Harrison, and Schaffner, 1♀ (00093269) (TAMU). **Guerrero**: Acahuizotla, 17.3833°N, 99.45°W, 944 m, 22 Jun 1982, L. Torres, 1♀ (00093270) (TAMU). **Jalisco**: El Molino, 20.12625°N, 103.14738°W, 1774 m, 10 Jul 1956, R. and K. Dreisbach, 1♀ (00070075) (USNM). **Michoacan**: 10.6 mi S of Uruapan, 18.96534°N, 102.10035°W, 24 Jul 1983, Kovarik, Harrison, and Schaffner, 1♂ (00093267) (TAMU). El Salitre, 20.16667°N, 102.66666°W, 1595 m, 29 Jul 1985, R. Barba, 1♂ (00094241), 1♀ (00094242) (IBUNAM). **Oaxaca**: 20 mi N of Putla, 17.40206°N, 97.60865°W, 2320 m, 03 Aug 1976, Peigler, Gruetzmacher, R. and M. Murray, Schaffner, 1♀ (00093268) (TAMU). **Sinaloa**: Santa Lucia, 23.49755°N, 105.92295°W, 1219 m, 16 Jul 1964, L.A. Kelton, 1♂ (00112960) (CNC); 04 Aug 1964, L.A. Kelton, 1♂ (00112934), 1♀ (00112953) (AMNH), 14♂ (00112917, 00112915, 00112921, 00112923–00112930, 00112932, 00112933, 00111000), 23♀ (00112922, 00112936–00112952, 00112955–00112959) (CNC), 1♂ (00112935), 1♀ (00112954) (USNM).

## Supplementary Material

XML Treatment for Ilnacora henryi
